# Nutritional management for a lung cancer patient receiving concurrent radiotherapy and immunotherapy and developing immune-related dermatitis: a case report

**DOI:** 10.3389/fnut.2025.1699651

**Published:** 2025-11-17

**Authors:** Yanlin Zeng, Xinmei Zhang, Di Qin, Yanping Zhong, Bihong Wei, Jun Liang

**Affiliations:** 1 Department of Radiotherapy, National Cancer Center/National Clinical Research Center for Cancer/Cancer Hospital & Shenzhen Hospital, Chinese Academy of Medical Sciences and Peking Union Medical College, Shenzhen, China; 2 Department of Nursing, National Cancer Center/National Clinical Research Center for Cancer/Cancer Hospital & Shenzhen Hospital, Chinese Academy of Medical Sciences and Peking Union Medical College, Shenzhen, China

**Keywords:** nutritional management, lung cancer, concurrent radiotherapy and immunotherapy, immune-related dermatitis, case report

## Abstract

**Introduction:**

Concurrent radiotherapy and immunotherapy is an alternative treatment regimen for elderly patients with locally advanced non-small cell lung cancer (NSCLC) who have contraindications to or decline surgery and chemotherapy. While these patients are susceptible to malnutrition and treatment-related adverse events, experience for their nutritional management is still lacking. In this case report, we summarized the nutritional management process for a NSCLC patient with severe malnutrition, who received concurrent radiotherapy and immunotherapy and developed immune-related dermatitis.

**Case summary:**

In March 2024, a 71-year-old male with lung squamous cell carcinoma and severe malnutrition was hospitalized in Cancer Hospital Chinese Academy of Medical Sciences, Shenzhen Center. During concurrent radiotherapy and tislelizumab immunotherapy, the patient developed grade 2 radiation-induced esophagitis and grade 3 immune-related dermatitis. The medical team dynamically assessed the patient’s nutritional status, set reasonable nutritional goals, and promptly adjusted nutritional prescriptions according to the severity and cause of the nutritional gap. Key measures included upgrading to combined enteral and parenteral nutrition support during radiation-induced esophagitis complicated by immune-related oral ulcers, adjusting the type of parenteral nutrition formulation for suspected lipid emulsion allergy, and strengthening immunonutrient supplementation during the dermatitis phase. Concurrently, progressive aerobic and resistance exercise training was guided to promote rehabilitation. Via the phased nutritional management, the patient’s nutritional indicators significantly improved after 54 days of hospitalization. Anti-tumor treatment was successfully completed, and the dermatitis healed.

**Conclusion:**

For lung cancer patients complicated with immune-related dermatitis during concurrent radiotherapy and immunotherapy, whole-course management including phased adjustment of nutritional strategies, early differentiation of dermatitis, and integration with exercise rehabilitation may effectively improve nutritional status and promote dermatitis healing, thus supporting the completion of anti-cancer therapy. Further studies with larger sample sizes are required to confirm the effect of nutritional management.

## 1 Introduction

Lung cancer is the most prevalent cancer type and the leading cause of cancer-related death (1). Locally advanced non-small cell lung cancer (NSCLC) is managed with various modalities including surgery, radiotherapy, chemotherapy, and targeted therapy, etc. In recent years, concurrent chemoradiotherapy (CCRT) followed by immunotherapy maintenance is recommended for patients with inoperable Pancoast tumors (2). Preliminary clinical studies suggest that for elderly patients with contraindications to or declining surgery and chemotherapy, concurrent radiotherapy and immunotherapy can be a safe and effective alternative (3). Approximately 15–40% of cancer patients suffer from malnutrition, with the incidence rising to 40–80% during treatment. Among lung cancer patients, 26.6–42.9% are at risk of malnutrition. Patients receiving chest radiotherapy may reduce oral intake due to radiation-induced esophagitis. Immunotherapy can indirectly lead to weight loss by causing nausea, diarrhea, etc. (4). Furthermore, about 5–10% of patients receiving immunotherapy develop severe dermatologic adverse events presenting as oral mucositis, which exacerbates nutritional deficiencies via reducing oral intake (5). The deterioration of nutritional status not only directly compromises treatment efficacy but also aggravates treatment-related toxicities, forming a vicious cycle ultimately impacting prognosis (4, 6). The role of nutritional support in cancer treatment is increasingly recognized. The European Society for Clinical Nutrition and Metabolism (ESPEN) guideline states that patients receiving radiation to the esophagus should undergo enhanced nutritional screening and intervention (7). However, concurrent radiotherapy and immunotherapy for lung cancer is still in the exploratory stage. Nutritional management strategies for this population is still unestablished, especially for those complicated by severe malnutrition and immune-related dermatitis.

Here we reported the treatment practice for a locally advanced NSCLC patient with severe malnutrition who developed grade 3 immune-related dermatitis during concurrent radiotherapy and immunotherapy, with emphasis on nutritional management. Through systematic nutritional assessment, phased nutrition interventions, nursing care, and exercise guidance, the patient’s dermatitis and nutritional status improved. He successfully completed this course of anti-tumor treatment before discharge.

## 2 Case information

**Figure 1:**
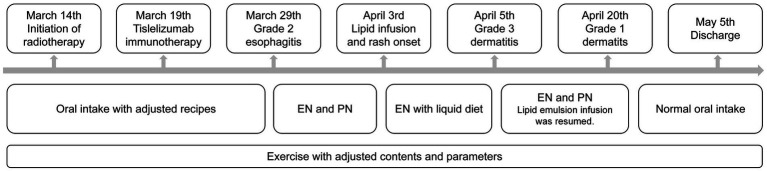
Timeline of clinical situation and support strategies. EN, enteral nutrition; PN, parenteral nutrition.

A 71-year-old Asian male was admitted to Cancer Hospital Chinese Academy of Medical Sciences, Shenzhen Center with the chief complaint “right chest and back pain for over 2 months, discovery of a right upper lung mass for 1 week.” His initial chest CT scan revealed suspicious cancer presented in the right upper lung, with a maximum diameter of 7.4 cm and invading the adjacent vertebral bodies and subclavian artery. The right hilar and mediastinal 4R lymph nodes were enlarged. Further image examinations including neck lymph node ultrasound, abdomen CT, brain MR and bone scanning excluded other lymph node and distal metastasis. After percutaneous aspiration biopsy of lung, he was diagnosed with right superior sulcus squamous cell carcinoma of stage cT4N2M0 IIIB (AJCC 9th edition). Neither family cancer nor allergic history was indicated. Nutritional screening revealed severe malnutrition. Because of his inoperability and possible intolerance to concurrent chemotherapy, multidisciplinary team (MDT) discussion was organized and led to the special decision of radiotherapy concurrent with immunotherapy. The radiotherapy was performed with 6MV-X rays and volumetric modulated arc therapy (VMAT) technique. The dosage prescription for the primary tumor was 25 Gy in 5 fractions, with the gross target volume receiving concurrent boost of 50 Gy. And 45 Gy in 15 fractions was prescribed to the lymph node metastasis. Immunotherapy with 200 mg tislelizumab was given 5 days after radiotherapy initiation. Two weeks later, the patient developed swallowing pain and preferred liquid diet. Blood test found the C-reactive protein rose to 21 mg/L but the procalcitonin remained negative. Based on the treatment history, symptoms and laboratory examinations, he was diagnosed with grade 2 radiation-induced esophagitis. During the first infusion of lipid emulsion, red rashes appeared with simultaneous respiratory symptoms. While other symptoms immediately relieved after low-flow oxygen support and discontinuation of lipid emulsion infusion, the rash progressively worsened and involved oral mucositis. Assessment suggested an immune-related adverse event (irAE), diagnosed as grade 3 immune-related dermatitis. Oral prednisone 30 mg daily and antihistamines were administered. Combined with whole-course nutritional management and skin care, the nutritional status improved, and the dermatologic manifestations subsided. The patient finally completed radiotherapy before discharged (see Figure 1).

## 3 Nutritional management

### 3.1 Nutritional risk assessment and initial nutritional prescription

A medical team consisting of oncology doctors, nurses and registered dietitians was responsible for whole-course nutritional management. Standardized nutritional screening was completed within 24 h of admission. His initial Nutrition Risk Screening 2002 (NRS 2002) score was 5, and Patient-generated Subjective Global Assessment (PG-SGA) score was 13, ranked Category C. Physical examinations indicated the height of 180 cm and weight of 58 kg, with the body mass index (BMI) of 17.9 kg/m^2^. His mid-upper arm and calf circumferences were 24 cm and 28 cm, respectively. And his handgrip strength was 19.3 kg. Laboratory tests of main nutritional indicators reported the albumin of 34.4 g/L, total protein of 70 g/L, and hemoglobin of 65 g/L. Collectively, the patient was diagnosed as severe malnutrition. Next, the doctor and dietitian design the initial recipe. According to nutritional guidelines for patients receiving radiotherapy (7), the recommended intake was at least 20–25 kcal/(kg·d) of energy and 1.5 g/(kg·d) of protein. As the patient was capable of performing simple physical activity, a high energy goal of 25 kcal/(kg·d) was set. Therefore, the individualized daily nutritional goals were 1,450 kcal of total calories and 87 g of protein. The patient performed 24-h dietary recall for several days, which revealed the average daily intake of 608 kcal energy and 33.45 g protein, reaching only 41.93 and 38.44% of the targets, respectively. His diet mainly consisted of carbohydrates like rice porridge, presenting a low-calorie, low-protein, and low-fiber pattern. Considering the nutritional gap and the patient’s ability to eat a regular diet, an initial full enteral nutrition plan was formulated. It was characterized by Nutrison (a standard enteral nutrition powder) combined with a high-protein regular diet (see Supplementary Table 1). Supply of Nutrison was started at 1/3 dose on the first day and reaching the full dose within 3 days to promote enteral nutrition tolerance.

### 3.2 Nutritional adjustment during radiation-induced esophagitis

During hospitalization, nurses recorded the daily intake to check the patient’s compliance to the recipe. And the dietitian performed weekly routine evaluation of nutritional status. Two weeks after anti-tumor treatment, the patient reported dysphagia and odynophagia. The symptoms progressed to influencing food consumption, together with the slight rise of inflammatory indicators, he was diagnosed with grade 2 radiation-induced esophagitis. Dietary recall over three consecutive days showed the oral intake was below 60% of requirement, with significant protein deficiency. Nurses reported this problem to both doctors and dietitians for timely reevaluation and adjustment. The PG-SGA score was 13, indicating a recurring nutritional gap. Because the patient refused nasogastric tube placement, combined enteral and parenteral nutrition support was promptly initiated. The enteral support was adjusted in food types and energy amount. An individualized diet plan combining soft, semi-liquid, and liquid foods was formulated based on the patient’s preferences to improve appetite. Total energy amount of the new diet plan was reduced according to the patient’s actual intake capacity (see Table 1). The remaining requirement was planned to be mainly supplemented via peripheral venous infusion of lipid emulsion and amino acids (18AA-II), aiming to cover the daily caloric gap of 699.19 kcal and protein gap of 45.34 g.

**Table 1:** Individualized nutrition recipe during grade 2 radiation-induced esophagitis.

Meal time	Foods	Weight (g)	Calories (kcal)	Protein (g)	Fat (g)	Carbohydrate (g)
Breakfast	Nutrison	6 scoops (30 g)	139.08	5.55	5.46	16.92
	Porridge	50	23	0.55	0.15	4.95
	Steamed egg custard	100	60.65	5.09	4.05	1.01
Lunch	Soft noodles	50	60	1.67	0.5	13
	Perch	50	52.5	9.3	1.7	0
	Tofu	100	84	6.6	5.3	3.4
Dinner	Porridge	50	23	0.55	0.15	4.95
	Minced meat	50	169.5	6.8	15.3	1.1
Snack	Nutrison	6 scoops (30 g)	139.08	5.55	5.46	16.92
Total	–	–	750.81	41.66	38.07	62.25

### 3.3 Early identification of immune-related dermatitis and comprehensive support

**Figure 2:**
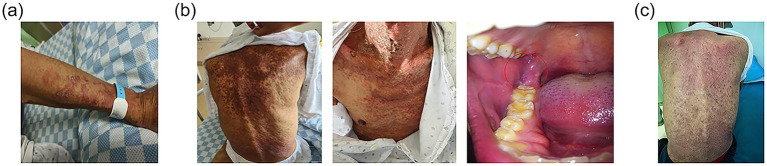
Development of immune-related dermatitis. **(a)** On the first day of lipid emulsion infusion, rash occurred on patient’s forearm. **(b)** Several days after stopping lipid emulsion supplement, the dermatitis still progressed to involving over 30% of body surface area, mainly presented on the chest and back. Moreover, oral mucositis (indicated with the red circle) developed. **(c)** On the day of discharge, the dermatitis recovered with residual pigmentation.

Upon the first infusion of lipid emulsion, the patient developed scattered rashes with pruritus, prominent on the upper limbs, accompanied by chest tightness and shortness of breath (see Figure 2a). Lipid emulsion infusion was urgently stopped, and low-flow oxygen support was given. Then the respiratory symptoms were alleviated soon. Considering the possibility of allergy to the peripheral venous formulation, he was switched to a full enteral liquid diet plan (see Table 2).

**Table 2:** Full enteral liquid diet during dermatitis onset.

Meal time	Foods	Weight (g)	Calories (kcal)	Protein (g)	Fat (g)	Carbohydrate (g)
Breakfast	Nutrison	11 scoops (55 g)	254.98	10.18	10.01	31.02
Snack	High-protein grain paste	~130	250	18	12	25
Lunch	Fruit and vegetable juice 1	~300	200	5	10	25
	Whey protein powder	20	80	16.48	0.5	1
Snack	Nutrison	11 scoops (55 g)	254.98	10.18	10.01	31.02
Dinner	Egg and tofu soup	~200	200	12	12	8
Snack	Fruit and vegetable juice 2	~150	100	1.2	5.5	11
	Nutrison	11 scoops (55 g)	254.98	10.18	10.01	31.02
Total	–	–	1594.94	83.22	70.03	152.06

All foods require thorough blending with a high-speed blender and fine sieving to achieve a residue-free, liquid consistency suitable for straw suction.

Homemade Recipes: (a) High-protein grain paste: 30 g oatmeal, 50 g chicken breast paste, 50 g pumpkin puree, 5 mL olive oil; (b) Fruit and vegetable juice 1: 100 g ripe banana, 100 mL carrot juice, 100 mL unsweetened soy milk, 5 mL flaxseed oil. (c) Egg and tofu soup: 1 egg, 50 g soft tofu, 150 mL vegetable clear broth, 5 mL sesame oil. (d) Fruit and vegetable juice 2: 100 g ripe apple, 100 mL spinach Juice, 100 mL milk, 5 mL olive oil.

However, the rash continued to worsen days after stopping parenteral nutrition. It was mainly presented on the chest and back, with more than 30% of body surface area affected. Moreover, oral mucosa was also involved (see Figure 2b). The nutritional management team together with doctors from the department of internal medicine discussed carefully and inclined to the diagnosis of grade 3 immune-related dermatitis. Thus, daily oral prednisone and loratadine were administered. Dietary recording revealed the patient could not consume the recommended amount due to the painful oral ulcer. Therefore, parenteral nutrition was prudently restarted.

There are significant differences in composition between fish oil-based lipid emulsion and standard lipid emulsion. The former is rich in *ω*-3 polyunsaturated fatty acids (PUFAs), while the latter mainly contains soybean oil and rich in ω-6 PUFAs. The fish oil-based product has stronger anti-inflammatory and immunomodulatory properties and is suitable for inflammatory conditions (7, 8). Thus, the parenteral nutrition regimen was adjusted to ω-3 PUFA-containing fish oil lipid emulsion, combined with 9AA compound amino acids. Total nutrient admixture with appropriate calorie-to-nitrogen and glucose-to-lipid ratios was also supplied. This regimen was successfully tolerated and maintained. Simultaneously, skin care measures including saline cleansing, hydropathic compress with skin lesion lotion, and topical recombinant human epidermal growth factor (rhEGF) gel were adopted. After over one week of anti-inflammatory treatment, parenteral nutrition support, and skin care nursing, the rash gradually subsided. Following comprehensive assessment of nutritional status and inflammatory markers, radiotherapy was continued, but immunotherapy was suspended.

### 3.4 Design and whole-course implementation of exercise program

Adequate diet and regular physical activity during treatment served as adjunctive therapy to improve prognosis. We designed an exercise program for the patient throughout the treatment course (see Supplementary Table 2). Through exercise combined with nutrition intervention, the patient’s exercise capacity improved, indicated by measurement of handgrip strength and 6-min walk distance (see Supplementary Table 3; Supplementary Figure 1).

### 3.5 Individualized discharge nutrition guidance

At discharge, the patient’s nutritional status was improved. Nutritional indicators including body weight, handgrip power, walking distance and laboratory markers increased, while PG-SGA score decreased compared to those at admission and during the adverse event period (see Supplementary Table 3; Supplementary Figure 1). His dermatitis recovered with residual pigmentation (see Figure 2c). The oral ulcers healed and the esophagitis partially relieved. With improved appetite, he gradually tolerated a regular diet and consumed enough nutrients orally. Based on the high-protein, anti-inflammatory, and antioxidant principles, a modified Chinese-Mediterranean diet pattern was guided by the dietitian. An individualized post-discharge diet plan was formulated (see Supplementary Table 4). Core foods containing protein sources included deep-sea fish, eggs, and tofu. Antioxidants were mainly composed of fruits like blueberries and kiwi. And anti-inflammatory foods were lipids like olive oil and walnut. During follow-up, the patient’s nutritional status remained stable, allowing for further anti-tumor treatment.

## 4 Patient perspective

The patient’s perspective was collected to evaluate the quality and defects of nutritional support. His reply was as follows: “With the daily care from the nutritional management team, I clearly understood what to eat and how to exercise. It was helpful to my nutritional status improvement and support me through the anti-cancer therapy. And it would be better if the team could offer food instead of only recipes, since some kinds of food was not easy to gain or process”.

## 5 Discussion

This patient had a superior sulcus NSCLC with surgical contraindications due to tumor invasion of surrounding critical anatomical structures. Relative contraindications to chemotherapy were present including the age and malnutrition. For elderly patients with locally advanced NSCLC who are inoperable or decline chemotherapy, preliminary clinical studies suggest concurrent radiotherapy and immunotherapy significantly improves survival prognosis compared to standalone radiotherapy or immunotherapy. The incidence of severe treatment-related adverse reactions (grade 3 or higher) resulting from this regimen does not significantly increase compared to single modalities (3, 9, 10). Therefore, after MDT discussion, this patient received current radiotherapy and immunotherapy. However, he developed grade 2 radiation-induced esophagitis and grade 3 immune-related dermatitis. A phase II clinical trial of locally advanced NSCLC patients treated with concurrent durvalumab and conventional fractionated radiotherapy reported severe adverse events mainly as lymphopenia and pneumonitis, while severe esophagitis and dermatitis were not common (9). In contrast, another study of early-stage NSCLC patients receiving stereotactic body radiotherapy (SBRT) concurrent with immunotherapy found that skin and mucosal toxicities like pruritus, rash, and oral mucositis were relatively common, including a case of grade 3 rash (11). In our case, the patient received hypofractionated radiotherapy. Similar to the SBRT trial, it delivered a relatively high radiation dose each fraction. This radiotherapy modality may induce distinct immune responses and being the cause of severe inflammation, though more research evidence is required to prove this hypothesis. Moreover, compared to the formerly mentioned trials, our case was presented with a poor baseline nutritional status, also being a possible contributing factor to severe adverse reactions. Future research with nutritional status stratification is warranted.

During the esophagitis phase, dietary strategy was adjusted from regular food to a combination of soft, semi-liquid, and liquid foods, and supplemented with oral nutritional supplements (ONS). Due to insufficient oral intake and refusal of tube feeding, the strategy was promptly escalated to combined parenteral nutrition, upon which the patient developed dermatitis. Lipid emulsion is a common parenteral allergen. Related allergic reactions often occur within 30 min of infusion initiation, with skin reactions typically presenting as urticaria, erythema, and pruritus. Severe cases may manifest as fever, chills, chest tightness, and palpitations, which usually resolve after discontinuation of lipid infusion and anti-allergy treatment (12). On the contrary, immune-related dermatitis typically occurs around 3 to 4 weeks after immunotherapy initiation. Common types include maculopapular rash, pruritus, psoriasis-like, and lichenoid lesions. Oral mucosa involvement is less common but has also been reported. Mild cases usually resolve after symptomatic treatment within 2 weeks, while severe ones require systemic corticosteroids or even biologics (5). As this patient’s dermatitis worsened after stopping lipid emulsion, together with the morphological features, area of involvement, and treatment response, we considered the diagnosis of immune-related dermatitis. Because of the simultaneous worsening of nutrition intake, restarting parenteral nutrition was deemed necessary and not absolutely contraindicated. We cautiously restarted parenteral nutrition with *ω*-3 PUFAs, which showed benefits in improving nutritional status and modulating inflammation in locally advanced NSCLC patients undergoing chemoradiotherapy (6). Combined with corticosteroid treatment and skin care, the patient’s immune-related dermatitis significantly resolved.

Beyond whole-course nutritional assessment and support, exercise plays a vital role in nutritional management. Exercise has been proposed to be a part of lung cancer treatment. It improves muscle strength, reduces muscle loss, and enhances overall status (13). Suitable exercises for lung cancer patients include aerobic and resistance training. Aerobic exercise enhances cardiopulmonary function, while resistance training is crucial for restoring muscle mass when combined with other exercises. Additionally, breathing exercise and flexibility training benefit cardiopulmonary function, fall prevention, and psychological state (14). However, specific exercise prescriptions require individualized design based on patient status. Temel et al. designed a structured exercise program consisting of aerobic exercise and weight training over an 8-week period for hospitalized advanced stage NSCLC patients. They found that those who completed the program experience improvement in cancer symptoms, but only less than half of the participants were able to complete the intervention (15). The NEXTAC-TWO trial offered nutritional and exercise treatment for elderly patients with advanced NSCLC, similar to the strategies for our case. However, no significant benefits were observed in the experiment arm even the patients had good compliance. One of the possible reasons is that their exercise program only includes low-intensity daily muscle training (16). For our case, a phased exercise prescription combining aerobic and resistance training with progressive exercise intensity effectively maintained and even improved his muscle strength. Implementing respiratory and flexibility training in such patients is a strategy worth trying in the future.

According to ESPEN guideline and other recent studies, specialist dietitians are essential in the multidisciplinary cancer nutritional management team (7, 17). In our practice, the basic team members include doctors and nurses from the department of oncology and dietitians. The doctor, nurse and dietitian evaluate the nutritional status together during the first visit. Then doctors and dietitians set the goal and design the individualized recipe. Dietitians are helpful in diet education as well. Nurses are responsible for daily consumption record, and dietitians perform reevaluation weekly. Doctors evaluate the cancer, treatment-related adverse events and any other medical problems influencing nutritional status. Once there was any nutritional event, the doctors, nurses and dietitians should alert each other and discuss for adjustment. This working pattern works effectively so far, but we are trying to include therapists from the department of rehabilitation so that we may refine the exercise guidance. Also, our team would benefit from recruiting members of food suppliers, based on the patient’s feedback.

In this case, systematic multi-dimensional nutritional assessment and dynamic nutritional support strategies were utilized based on treatment phase and complication characteristics. Addressing the specific needs of immune-related dermatitis, immunonutrients like *ω*-3 fatty acids were integrated into the nutritional plan. Combined with exercise management and nursing care, the effectiveness of nutritional interventions were ensured. To our knowledge, as concurrent immunotherapy and radiotherapy is not a routine strategy for lung cancer treatment, this case is the first report providing nutritional support experience for patient with this treatment background. However, there are several limitations of this case report. First, as a single case report, the results should be interpreted and generalized with caution. Second, due to the patient’s moving away, we were unable to provide formal data of long-term follow-up on nutritional status and cancer outcomes. Third, the reproducibility of the combined exercise and nutrition program in routine clinical practice outside our center is uncertain because of the possible different designs and management. In summary, the optimal nutritional management strategy for such patients still requires further research exploration.

## 6 Conclusion

Whole-course nutritional management integrated with exercise effectively improved nutritional status for a lung cancer patient complicated with immune-related dermatitis during concurrent radiotherapy and immunotherapy. Future clinical research was warranted to generalize the results of this case.

## Data Availability

The original contributions presented in the study are included in the article/Supplementary material, further inquiries can be directed to the corresponding author.
