# Supplementary material for: Nutritional management for a lung cancer patient receiving concurrent radiotherapy and immunotherapy and developing immune-related dermatitis: a case report
Source: Front Nutr. 2025 Nov 17;12:1699651. doi: 10.3389/fnut.2025.1699651 (PMC12667434; doi:10.3389/fnut.2025.1699651)
Supplement: Supplementary file 1 [file Data_Sheet_1.docx]

Nutritional management for a lung cancer patient receiving concurrent radiotherapy and immunotherapy and developing immune-related dermatitis: a case report

**Supplementary Table 1. Core components of initial full enteral regular diet nutrition plan**

| Meal time | Core foods | Weight (g) | Calories (kcal) | Protein (g) |
| --- | --- | --- | --- | --- |
| Breakfast | Egg | 50 | 72 | 6.9 |
|  | Nutrison | 10 scoops (50g) | 231.83 | 9.25 |
| Snack | Yogurt | 100 | 62 | 3.2 |
| Lunch | Steamed Perch | 100g | 97 | 18.6 |
|  | Soft noodles | 150 | 180 | 5 |
|  | Olive oil | 5mL | 45 | 0 |
| Snack | Nutrison | 3 scoops (15g) | 69.54 | 2.775 |
|  | Apple juice | 100ml | 46 | 0.1 |
| Dinner | Chicken breast | 100 | 165 | 24.6 |
|  | Nutrison | 10 scoops (50g) | 231.83 | 9.25 |
| Snack | shrimp paste | 100g | 99 | 23 |
|  | Nutrison | 10 scoops (50g) | 231.83 | 9.25 |
| Total | - | - | 1531.03 | 106.92 |

**Supplementary Table 2. Exercise program**

| Week | Training type | Training content and parameters | Completion status | Vital signs | Adverse effects |
| --- | --- | --- | --- | --- | --- |
| Week 1 | Resistance | Ankle pump: Dorsiflexion/Plantarflexion (5 sec/rep)  Sets: 15 reps/set × 3 sets  Rest between sets: ≤1 min | Completion 100% | HR: 85-100 bpm  SpO₂: 96-99% | None |
|  | Aerobic | Indoor level walking Speed: 1.5-2.0 km/h  RPE: 9-11  Duration/Session: 20 min  Frequency: 2-3 times/day | Mid-session break once | HR: 85-100 bpm  SpO₂: 96-99% | None |
| Week 2 | Resistance | Maintain ankle pump (Same parameters as Week 1) | Completion 100% | HR: 85-100 bpm  SpO₂: 96-99% | None |
|  | Aerobic | Continuous walking in ward corridor  Speed: 2.0-2.5 km/h  RPE: 10-12  Duration/Session: 30 min  Frequency: 3-4 times/day  Requirement: ≤1 break/session | Uninterrupted (RPE 11) | HR: 90-105 bpm SpO₂: 95-98% | Day 3: Calf soreness (relieved after 10 min massage) |
| Week 3 | Resistance | Paused | - | - | - |
|  | Aerobic | Combined Walking:  Ward Walking (2.5 km/h)  Hospital Ground Level Walking (2.5-3.0 km/h)  Combined RPE: 12-13  Duration/Session: 30 min  Frequency: 3-4 times/day | Mild sweating during hospital walk (RPE 13) | HR: 100-115 bpm  SpO₂: 94-97% | None |
| Week 4 to Discharge | Resistance | Paused | - | - | - |
|  | Aerobic | Advanced Walking:  Ward Brisk Walking (3.5-4.0 km/h, RPE 13-14)  Hospital Walking (3.0 km/h)  Duration/Session: 30 min Frequency: 3-4 times/day | Mild sweating during hospital walk (RPE 13, target 14 not reached) | HR: 100-115 bpm  SpO₂: 94-97% | None |

Note:

RPE (Rating of Perceived Exertion, Borg Scale 6-20): Higher numbers indicate greater perceived effort. E.g., 9-11 (Very Light to Light), 12-13 (Somewhat Hard), 14 (Hard).

**Supplementary Table 3.** **Nutrition indicator monitoring**

| Week | PG-SGA score | Weight (kg) | Handgrip (kg) | Serum albumin (g/L) | Serum total protein (g/L) | Hemoglobin (g/L) | 6-min walk distance (m) |
| --- | --- | --- | --- | --- | --- | --- | --- |
| Week 1 | 13 | 58.0 | 19.3 | 34.4 | 65.7 | 65 | 210 |
| Week 2 | 10 | 58.5 | 20.1 | 36.6 | 65.7 | 87 | 233 |
| Week 3 | 12 | 58.6 | 20.7 | 33.5 | 59.8 | 87 | 252 |
| Week 4 | 14 | 58 | 20 | 32.5 | 59 | 86 | 200 |
| Week 5 | 10 | 58.3 | 21.6 | 33 | 57.7 | 90 | 250 |
| Week 6 | 9 | 58 | 23.2 | 38.1 | 58.7 | 97 | 310 |
| Week 7 | 5 | 58.5 | 24.0 | 40.2 | 72.3 | 117 | 350 |
| Improvement | -62.5% | +0.9% | +24.35% | +18.24% | +10.04% | +80% | +66.67% |

**Supplementary Table 4. Modified Chinese-Mediterranean diet plan**

| Meal | Foods | Weight (g) | Key nutrients |
| --- | --- | --- | --- |
| Breakfast | Multigrain porridge | 100 | 23 g carbohydrates |
|  | Steamed egg custard | 100 | 12 g protein |
|  | Spinach with olive oil dressing | 100 | 380 μg vitamin K |
| Snack | Whey protein powder with ω-3 fatty acid addition | 30 | 24 g protein and 1.2 g ω-3 fatty acid |
| Lunch | Steamed Perch | 150 | 27 g protein |
|  | Broccoli fried with mushrooms | 200 | 4 g dietary fiber |
|  | Multigrain rice | 100 | 35 g carbohydrates |
| Dinner | Tofu and shrimp soup | 150 | 18 g protein |
|  | Steamed pumpkin | 100 | 890 μg β-carotene |
|  | Purple cabbage | 100 | 26 mg vitamin C |

**
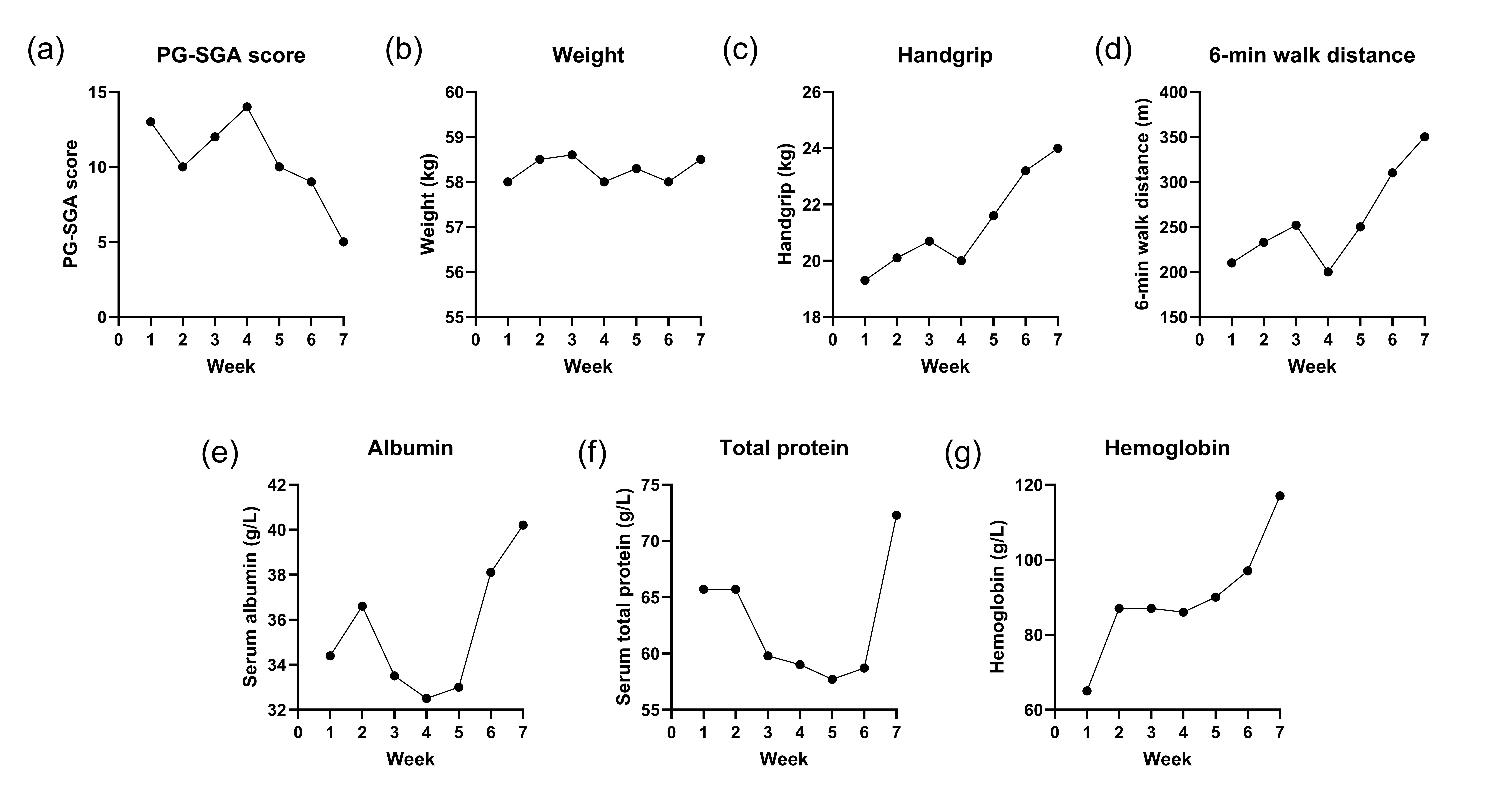
**

**Supplementary Figure 1. Trends of nutritional indicators**

Nutritional indicators were monitored every week during the whole hospitalization period. The main indicators included: (a) score of PG-SGA assessment, (b) body weight, (c) power of handgrip, (d) walking distance in 6 minutes, and laboratory examinations like (e) serum albumin, (f) serum total protein, and (g) hemoglobin.

Abbreviations: PG-SGA, Patient-generated Subjective Global Assessment.
